# Identification of Biomarkers for Type 2 Diabetes and Its Complications: A Bioinformatic Approach

**Published:** 2007-12

**Authors:** Srinubabu Gedela, Allam Appa Rao, Narasimha Rao Medicherla

**Affiliations:** *International Center for Bioinformatics and Center for biotechnology, College of Engineering, Andhra University, Visakhapatnam, India*

**Keywords:** type 2 diabetes, biomarkers, neuropathy, nephropathy, insulin resistance, bioinformatics

## Abstract

The long asymptomatic period before the onset of chronic diseases presents opportunities for disease prevention. Many chronic diseases like type 2 diabetes and its complications may be preventable by avoiding factors that trigger the disease process (primary prevention) or by use of therapies that modulate the disease process before the onset of clinical symptoms (secondary prevention). Accurate prediction and identification using biomarkers will be useful for disease prevention and initiation of proactive therapies to those individuals who are most likely to develop the disease. Recent technological advances in genetics, genomics, proteomics, and bioinformatics offer great opportunities for biomarker discovery. In this review, type 2 diabetes and its complications are used as examples discuss pertinent issues related to high throughput biomarker discovery using bioinformatic pathways.

## INTRODUCTION

Bioinformatics emerged first and laid the groundwork for proteomics and metabolimics with genome sequencing projects, microarray-based expression profiles (1) and phenotypic profiles at the cell and organismal levels (2). While bioinformatics focuses on the level of gene expression, proteomics and metabolomics study more direct biological insights into the function of the cell by measuring the expression levels of proteins and metabolites (3). Bioinformatics, therefore, provides the necessary data as to the gene expression and formulates further biological questions that proteomics and metabolimics try to address. All of the above fields are heavily dependent on experimental methods and instrumentation for high-throughput data collection and analysis required to accomplish the ambitious goals of analysis on a cell wide basis. Experimental methods such as microarray technology, liquid chromatography tandem mass spectrometry, and nuclear magnetic resonance profiling of metabolites make it feasible to address the biological questions of the “bioinformatics” era.

Along with instrumentation, computational methods have evolved to process the large quantities of data and to draw meaningful scientific conclusions. It is in this area of data analysis and computational method development that our research interests lay. We have reported some of the proteins revealed by bioinformatic analyses which are expected to be useful as biomarkers for diabetes and its complications. Such bioinformatic analysis will be useful for liquid chromatography tandem mass spectrometry data correlation with *in silico-simulated* data to identify peptides and proteins that are differentially expressed between biological samples derived from normal individuals and those taken from patients suffering from diabetes and its complications.

With many genomes sequenced, their respective transcriptomes can be predicted with a certain degree of accuracy. Genome-wide gene expression analysis (transcriptomics) can therefore deliver a comprehensive view on all genes active at a given time in a given sample. This degree of completeness in terms of analysis can be achieved with proteomics, bioinformatics or metabolimics, simply because neither the (e.g. human) proteome nor the metabolome are known. Consequently, bioinformatics is suitable for a first “round of discovery” in regulatory networks and serves to put proteomic and metabolomic markers into a larger biological perspective.

### Role of Metabolimics in Diabetes

The best approach to functional genomic analysis of an organism must include measurements of chemical constituents-the metabolites-of individual cells or tissues of the organism. By coupling metabolite analysis with the information provided by genomics, we can create a science of metabolic genomics and by combining this with information from transcriptomics and proteomics we come that much closer to fully deciphering the complex inner workings of living systems.

Akin to the genome, transcriptome and proteome, the metabolome is the entire set of metabolites synthesized by a biological system. Metabolimics is rooted in metabolite profiling, a term first coined in the 1970s to refer to the qualitative and quantitative analysis of complex mixtures of physiological origin (4). Recent technological advances allow researchers to separate and detect small molecules with astonishing sensitivity and selectivity. Depending on the scope and nature of the technique, the analysis of metabolites is termed target analysis, metabolic profiling, metabolimics, or metabolic fingerprinting. Target analysis is constrained to the quantitation of a single analyte and can be used to directly study the primary effect a genetic alteration. Because all other metabolites are incidental in this type of analysis, extensive sample clean-up may be required to avoid interference from other matrix compounds. Target analysis is principally utilized for screening purposes or in situations requiring extreme sensitivity. One step up from target analysis is metabolic (or metabolite) profiling in which the analysis is expanded to a number of pre-selected metabolites. Usually, the metabolites under analysis are part of one metabolic pathway or intersecting pathways. The medical community has used this type of metabolite analysis for many years as a screening and diagnostic tool, with monitoring of blood glucose and cholesterol levels being examples of metabolic profiling.

### Diabetes Mellitus

Diabetes mellitus is a disease of abnormal glucose metabolism resulting in hyperglycemia due to either a deficiency of insulin secretion or insulin resistance or both. Classic signs and symptoms of diabetes include polyuria, polydipsia, polyphagia, weight loss, headache, tachycardia, palpitations, and blurred vision (5). The diagnostic criteria for determining diabetes have recently been changed in order to increase the sensitivity of the test. Currently, diabetes is diagnosed by a fasting glucose of 126 mg/dl or greater on more than one occasion or a random glucose of 200 mg/dl or greater on any occasion (6). Impaired fasting glucose (IFG) is defined as a blood sugar of 100-125 mg/dl (5.6-6.9 mmol/l) (6). Impaired glucose tolerance (IGT) is defined as an abnormal 2-hour postprandial blood sugar of 144-199 mg/dl (6).

IFG and IGT comprise the category now known as ‘prediabetes’ (5). These relatively ‘new’ criteria differ from the previous diagnostic criteria established in 1985 by the World Health Organization (WHO) (7). The WHO criterion recommended diagnosis of diabetes with a single random blood sugar greater than 200 mg/dl (11.1 mmol/l) and the use of a 75 g oral glucose challenge test (OGTT) to diagnose those in “the uncertain range” (blood sugars 140-199 mg/dl). It is important to note that children with diabetes usually present with acute signs and symptoms, including coma or loss of consciousness, critical glucose levels, ketonemia, and marked glucosuria and ketonuria. The diagnosis in children is made immediately (rather than repeating a blood sugar).

Previously, the diagnosis of diabetes was made if the 2-hour post glucose load value was ≥200 mg/dl. Impaired glucose tolerance was diagnosed with OGTT values of <140 mg/dl fasting and a 2-hour post glucose load value of 140-199 mg/dl. In 1999, the WHO proposed changes in this system, decreasing the fasting plasma glucose diagnostic value to ≥126 mg/dl (on two separate occasions) as well as an OGTT value of 2-hour post challenge glucose of ≥200 mg/dl in an asymptomatic person (8). The DECODE (Diabetes Epidemiology: Collaborative Analysis of Diagnostic Criteria in Europe) Study analyzed the population impact of the changing criteria (DECODE Study Group European Diabetes Epidemiology Group, 1999). This study examined the results of population-based studies of blood sugars in the elderly and all causes of mortality. The study concluded that 33% of those who were undiagnosed at baseline had isolated post OGTT hyperglycemia. The group with isolated post OGTT hyperglycemia had similar risk of mortality as those diagnosed with diabetes. They concluded that the administration of an OGTT to those with impaired fasting glucose would increase the number of elderly diabetics by 50% (9).

Diabetes mellitus is a significant public health problem in the United States. According to the National Center for Chronic Disease Prevention and Health Promotion over 21 million Americans (7% of the population) have diabetes and 18 million people have prediabetic conditions (10). The World Health Organization (WHO) states that diabetes is “a world-wide epidemic” (11) and has devoted resources in screening for diabetes as well as primary prevention in order to reduce the significant impact of the diagnosis of diabetes and reduce its complications. The WHO estimates that over 30 million Americans will be diagnosed with incident diabetes in the year 2030 (World Health Organization, n.d.).

Recently we reported an analysis of genes causing hypertension, cardiovascular and diabetic diseases using a composition alignment method utilizing a new approach for analyzing DNA sequences to detect regions of similar nucleotide composition (12). Bhramaramba *et al* reported analysis of species affected by diabetes, which will be useful for protein folding studies (13). Bases on the serum butyrylcholinesterase levels (14) or brain derived neurotrophic factor (15), we may identify type 2 diabetes based on the cholinesterase levels, and may identify diabetes associated alzheimer’s disease (16). These enzymes are excepted to be useful as biomarkers for type 2 diabetes.

### Complications

**Figure 1:**
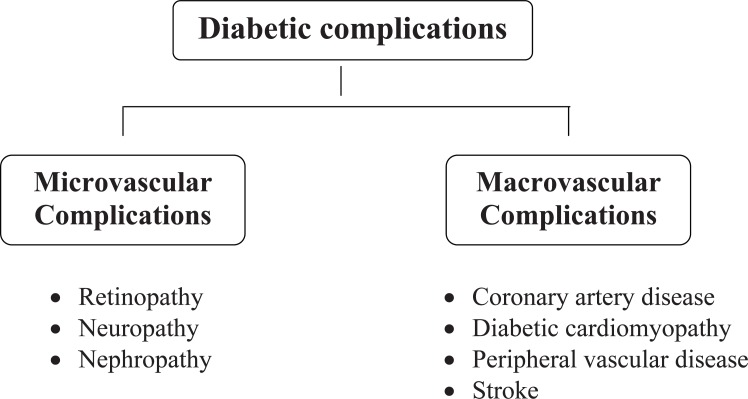
Diabetic complications.

Complications of diabetes are due to pathologic changes that involve small and large blood vessels, cranial and peripheral nerves, the skin, and the lens of the eye. Macrovascular complications involve damage to the large blood vessels of the brain, heart, and extremities. Microvascular complications of diabetes include retinopathy and nephropathy and are thought to be a result of an abnormal thickening of the basement membrane of the capillaries (Figure 1).

### Diabetic Retinopathy

Diabetic retinopathy consists of microaneurysms, hemorrhages, exudates, and retinal edema, as well as proliferation of newly formed vessels in some cases. Retinopathy may result in the loss of vision. Diabetic retinopathy is a highly specific vascular complication of both Type-I and Type-II diabetes. The prevalence of retinopathy is strongly related to the duration of diabetes. After 20 years of diabetes, nearly all patients with type I diabetes and >60% of patients with Type II diabetes have some degree of retinopathy. It is the leading cause of blindness in the western world.

In general, the progression of retinopathy is orderly, advancing from mild non-proliferative abnormalities, characterized by increased vascular permeability, to moderate and severe non-proliferative diabetic retinopathy (NPDR), characterized by vascular closure, to proliferative diabetic retinopathy (PDR), characterized by the growth of new blood vessels on the retina and posterior surface of the vitreous(37). Divakar *et al* reported a computational protein sequence analysis for diabetic retinopathy (38), and Rao et el reported a bioinformatic analysis of diabetic retinopathy (39). Wilkinson-Berka (49) reviewed the role of the renin–angiotensin system in diabetic retinopathy and the potential of its blockade as a treatment strategy for this vision-threatening disease.

Large clinical trials have emphasized that blood pressure control provides a major clinical benefit in reducing the risk of blindness in patients with diabetic retinopathy (50). Retinal neovascularization in diabetes has been thought to follow the release of local angiogenic factors in the retina. We hypothesize that neovascularization of diabetic retinopathy represents systemic vasculogenesis rather than local angiogenesis. Thus, we evaluate the concentrations of circulating endothelial progenitor cells (EPCs) and stem cell modulation factors such as vascular endothelial growth factor (VEGF), erythropoietin (Epo), and substance p (SP) in the peripheral blood of diabetic retinopathy patient.

### Diabetic Neuropathy

Diabetic neuropathy may involve either the periphery, gastrointestinal, genitourinary, or all systems. Diabetic neuropathy produces symptoms in 60-70% of all diabetic persons. Neuropathic complications are divided into autonomic dysfunction and sensory dysfunction. Sensory complications include paresthesias and the loss of sensation in the extremities, leading to an increase in serious foot problems in diabetics. Autonomic complications include sexual dysfunction, gastrointestinal disturbances, bladder dysfunction, and postural hypotension (26). We have reported bioinformatic analysis of diabetic neuropathy using functional protein sequence (27). The recent development of bioinformatic analysis has made it feasible to analyse protein profiles in various cells, tissues and body fluids with only a small sample (28). However, few proteome analyses in human vitreous fluid have been performed in the setting of diabetic eye disease (29-32). The nephron filters fluid from the blood and converts it to urine. Recently Leinninger (51) discussed the proposed role of mitochondrial degeneration in the pathogenesis of diabetic neuropathy, and highlight potential mitochondrial sites for therapeutic intervention.

### Diabetic Nephropathy

Diabetic nephropathy is a serious microvascular complication of diabetes. Diabetes mellitus is the most common cause of end-stage renal disease in the United States (17). The risk factors for nephropathy are older age, male sex, non-Caucasian race, and poor blood pressure, glycemic, and lipid control. The kidneys have several important functions: excreting waste, maintaining blood pressure through the regulation of fluid and salts, production of erythropoietin (a regulator of red blood cell mass), and activation of vitamin D (a co-factor for calcium absorption). Normal kidney function involves the filtration of fluid from the blood and formation of urine.

The early pathogenesis of diabetic nephropathy begins with hyperglycemia causing glomerular hyperfiltration, which results in glomerular hypertrophy and glomerular basement membrane thickening. Early nephropathy also involves hemodynamic changes, including, decreased afferent and efferent arteriolar resistance, a dramatically increased plasma flow, and a moderately increased glomerular capillary pressure leading to an increased glomerular filtration rate (GFR). Subsequently, the GFR declines (18). The GFR is defined as the volume of plasma that can be completely cleared of a particular substance by the kidneys in a unit of time. The gold standard for determining GFR has traditionally been inulin or iohexol but these techniques are invasive, expensive and time-consuming (19).

One of the early signs of diabetic nephropathy is the presence of microalbuminuria. Microalbuminuria is defined as an albumin excretion rate 20-200 μg/min (30-300 mg/dl) or a urinary albumin to creatinine ratio of 2.5-35 mg/mmol in men and 3.5-35 mg/mmol in women. In normal people (without diabetes), urinary albumin excretion rarely exceeds 10 μg/min. Eventually, the kidneys become ‘leaky’ and allow increased protein excretion, as well as red blood cells and casts. Diabetic nephropathy occurs in 30-40% of type 1 diabetics after 20 years and in 15-20% of type 2 diabetics (American Diabetes Association, 2003a). Diabetic nephropathy is initially manifested by proteinuria and as kidney function declines, urea and creatinine accumulate in the blood. Thus, an important method of detecting small amounts of urinary albumin is to analyze urine for microalbumin. A ‘spot’ microalbumin (a random sample) of <30 μg/mg creatinine is considered normal (20). Progressive diabetic nephropathy eventually leads to renal failure.

Diabetic nephropathy is first characterized by glomerules hemodynamic abnormalities that result in glomerular hyperfiltration, leading to glomerular damage as evidenced by microalbuminuria. As glomerular function continues to decline, overt proteinuria, decreased GFR and end stage renal failure will result (21). Recenlty, we have reported angiotension II and chemokines may be useful for diabetic nephropathy identification (22). The assessment of albumin excretion rate (AER) is currently the best available noninvasive method for the early recognition of pending renal disease in nonproteinuric diabetic patients; a controversy debate exists about its sensitivity and specificity in the current literature (23-25).

Greater efforts are urgently needed to properly screen for and diagnose diabetic nephropathy early in order to prevent long-term complication. Patient-education, dietitian-involvement and effective referral systems are issues that need attention in the primary care setting. Programs that both motivate patients to make the important but difficult lifestyle changes, and empower them to promote self-care, need to be initiated throughout the world.

### Macrovascular Complications

Cardiovascular complications are perhaps the most significant sequelae of diabetes mellitus. The National Cholesterol Education Project (NCEP) considered the presence of diabetes to be equivalent to the presence of coronary heart disease (33). 65% of all diabetics will die from cardiovascular or cerebrovascular disease (34). Nearly 45% of all diabetics have peripheral vascular disease. The increased risk of all types of vascular disease is most likely due to the high circulating insulin levels which have been theorized to stimulate the atherogenic process by inducing smooth muscle cell proliferation and cholesterol synthesis (35). The Helsinki Policeman Study was one of the first studies to link elevated plasma insulin levels to adverse cardiovascular disease outcomes in a healthy (nondiabetic) population. This cohort study consisted of 982 men ages 35-64 and found that serum insulin levels were predictive of CVD risk independent of other risk factors. Specifically, the incidence of cardiovascular events was four to five times higher among men in the highest insulin quintile compared to men in the lowest insulin quintile (36). Additionally, the diabetic state has been shown to alter platelet function, increase platelet aggregation and increase levels of fibrinogen-all conditions that promote clot formation in the vasculature, a major step in the pathogenesis of cardiovascular disease.

### Coronary Artery Disease

Coronary artery disease is the most common cause of death in adults with diabetes mellitus. DM is an independent risk factor for coronary artery disease and the incidence of coronary artery disease is related to the duration of diabetes. In patients with DM, myocardial infarctions are not only more frequent but also tend to be larger in size and more likely to result in complications such as heart failure, shock and death. Diabetic patients are more likely to have an abnormal or absent pain response to myocardial ischemia, probably as a result of generalized autonomic nervous system dysfunction. Ambulatory electrocardiographic monitoring has shown that upto 90% of episodes of ischemia are silent in diabetic patients with coronary artery disease. Other risk factors for coronary artery disease are hypertension, smoking and hyperlipidemia.

### Diabetic Cardiomyopathy

Heart failure can occur in patients with DM in the absence of coexistent hypertension and / or coronary artery disease. Clinical reports in 1970’s first described such patients, who were considered to have a diabetic cardiomyopathy, usually of dilated cardiomyopathy with low ejection fraction. Diabetic animals show impaired myocardial contractility, possibly related to decreased calcium pump activity in the sarcoplasmic reticulum. Diabetic cardiomyopathy may cause left ventricular dysfunction, detectable by echocardiography in asymptomatic diabetic subject, which may progress to severe left ventricular failure (47).

### Metabolic Syndrome

Metabolic syndrome, or insulin resistance syndrome or Syndrome X, is a syndrome of dyslipidemia, insulin resistance, central obesity, and hypertension with or without hyperglycemia (40). This syndrome is more common in certain ethnic groups including African Americans, Mexican Americans, Asian Americans and Pacific Islanders. Currently, the World Health Organization recommends the following criteria for diagnosis: impaired glucose metabolism, insulin resistance, blood pressure ≥140/90 mmHg, triglycerides ≥150 mg/dl and/or HDL cholesterol <35 mg/dl in men or <39 mg/dl in women, central obesity (waist to hip ratio in males >0.90 and >0.85 in women and/or BMI >30 kg/ m^2^), and microalbuminuria (urinary albumin excretion rate ≥20 μg/min or albumin:creatinine ratio ≥30 mg/g). Those with metabolic syndrome are at increased risk of development of cardiovascular complications, renal disease, and mortality (40-41). The Miami Community Health Study was a cross-sectional study performed in multi-ethnic Dade Count, Florida (42-43). The participants were 494 healthy African American, Cuban American, and non-Hispanic whites aged 25-44 years. The participants completed questionnaires on lifestyle and health habits and then had a clinical exam consisting of an EKG, anthropometric measurements, and physical measurements. This study was one of the first to include Hispanics.

In several published articles, Donahue *et al*. examined the effects of gender and ethnicity on body fat measures and insulin response, as well as the relation between blood pressure and fasting insulin levels. They found a positive association between fasting insulin levels and blood pressure in non-Hispanic whites and African-Americans and higher insulin area under the curve (AUC) in the ethnic groups compared to the non-Hispanic whites. Donahue *et al*. (42) also examined the association between the characteristics of insulin resistance syndrome (hyperlipidemia, hypertension, glucose intolerance and an android pattern of fat distribution) and the rate of insulin-mediated glucose disposal, a measure of insulin resistance, as well as fasting insulin levels. They found an association between uric acid, diastolic and systolic blood pressure, triglyceride levels, and waist circumference and a negative association with HDL levels and the rate of insulin-mediated glucose disposal. Unfortunately, this was a cross-sectional study associations could be made but causation could not be established (42-43).

Several studies have suggested that type 2 diabetes is an inflammatory condition, mainly due to finding increased levels of inflammatory markers such as C-reactive protein and IL-6 in type 2 diabetics. The endothelial function marker e-selectin has been shown to be increased in type 2 diabetes and to predict cardiovascular disease. The Nurses’ Health Study found that increased levels of E-selectin predicted type 2 diabetes, however the sample population consisted primarily of women. Numerous research studies have demonstrated that microalbuminuria is a predictor of cardiovascular disease and mortality. Researchers have discovered a new renal marker, cystatin C, as an accurate determinant of glomerular filtration rate. A few recent studies have found that cystatin C levels predict coronary artery disease and congestive heart failure. However, little research has been done to describe the relationship between the cystatin C and type 2 diabetes. Gumpeny *et al* reported the computer aided prediction of active site of enzymes dor diabetic metabolic syndrome (44).

When insulin was introduced into clinical practice, it was assumed that it would provide a complete therapy for diabetes mellitus. However, 50 years after the discovery of insulin, diabetic patients still have a considerably reduced life expectancy despite a significant reduction in the incidence of ketoacidosis. This excess mortality is mainly due to long term complications that affect the blood vessels, eyes, kidneys, heart and nerves. An understanding of the pathogenesis of diabetic complications will help in identifying potential sites of therapeutic intervention. (45). People with diabetes are 25 times more likely to develop kidney disease, 30-40 times more likely to undergo a major amputation, 2-4 times more likely to develop a myocardial infarction and twice as likely to suffer a stroke than individuals without diabetes (46).

**Figure 2:**
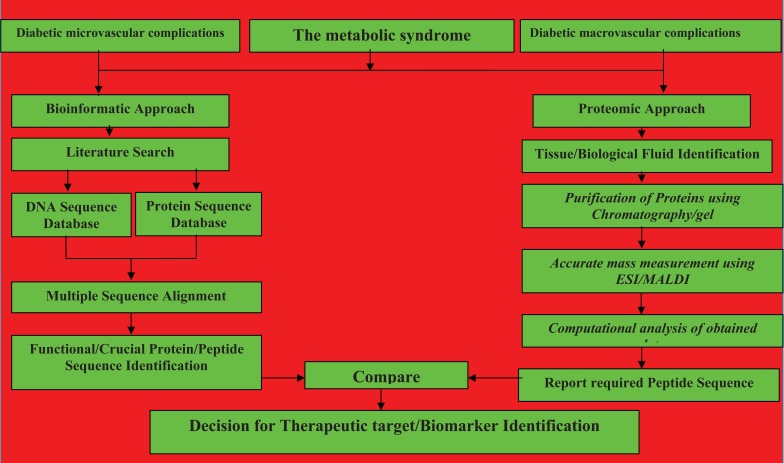
Schematic representation of the diabetic complications with reference to bioinformatic and proteomic approaches for therapeutic drug target identification and/or biomarker identification.

A schematic representation of the diabetic micro vascular complications with reference to bioinformatic and proteomic approaches for therapeutic drug target identification and/or biomarker identification is presented (Fig 2). Reported proteins using bioinformatic tools are expected to be useful as biomarkers and further experimental studies for the same using proteomic techniques will be useful.

## CONCLUSION & PERSPECTIVES

This review highlights the importance of identifying biomarkers for diabetes and its complications, and their impact on the healthcare system. Recent reports indicated that both inflammatory markers and endothelial function markers are increased in diabetes. In silico studies have estabilished a pathway for *in vivo/in vitro* studies for identification of proteins, having therapeutic significance. In the future therapeutic strategy targeting, target drug delivery systems and biomarker identification using the proteomic tools such as mass spectrometric analysis, microarray analysis are excepted to solve our hypothesis.
